# Total Parathyroidectomy With Brachialis Muscle Autotransplantation in a Renal Transplant Recipient With Tertiary Hyperparathyroidism: A Case Report

**DOI:** 10.7759/cureus.95969

**Published:** 2025-11-02

**Authors:** Maria Tzoraki, Dimitrios Tranoudakis, Ilias Karaiskos, Constantine Tsigos, Ioannis Giatras

**Affiliations:** 1 Department of Medical Services, Hygeia Hospital, Athens, GRC; 2 Department of General Surgery, Hygeia Hospital, Athens, GRC; 3 Department of 1st Internal Medicine and Infectious Diseases, Hygeia Hospital, Athens, GRC; 4 Department of Endocrinology, Diabetes, and Metabolism, Hygeia Hospital, Athens, GRC; 5 Department of Nutrition and Dietetics, School of Health Science and Education, Harokopio University, Athens, GRC; 6 Department of Nephrology, Hygeia Hospital, Athens, GRC

**Keywords:** brachialis muscle, parathyroid autotransplantation, parathyroidectomy, parathyroid gland adenoma, tertiary hyperparathyroidism

## Abstract

Tertiary hyperparathyroidism (THPT) is a complication of long-standing secondary hyperparathyroidism (SHPT) in patients with end-stage renal disease (ESRD), which may persist even after renal transplantation. It is defined by autonomous, dysregulated parathyroid hormone (PTH) secretion, often refractory to pharmacologic therapy, and typically necessitating surgical intervention. We report the case of a 72-year-old renal transplant recipient with persistent THPT who underwent total parathyroidectomy (tPTX) with selective autotransplantation of parathyroid tissue into the left brachialis muscle. This approach was chosen to address ectopic glands and anatomical complexity, while facilitating postoperative monitoring and preserving superficial forearm structures for potential future dialysis access. Postoperatively, PTH normalized at 10 months (57 pg/mL) with stable calcium and renal function, confirming durable graft function and clinical remission. Total parathyroidectomy with brachialis muscle autotransplantation provides a safe, technically feasible, and functionally advantageous option for managing advanced or ectopic THPT. To our knowledge, this represents the first documented case of brachialis autotransplantation for THPT in Greece.

## Introduction

Tertiary hyperparathyroidism (THPT) emerges from chronic secondary hyperparathyroidism (SHPT) in patients with longstanding end-stage renal disease (ESRD), often evolving into autonomous parathyroid hormone (PTH) secretion, which persists in approximately 20-30% of cases, despite renal transplantation [1, 2]. Unlike SHPT, THPT is characterized by loss of calcium-PTH feedback regulation [3], leading to sustained hypercalcemia, osteodystrophy, vascular calcifications, and, in some cases, calciphylaxis [4, 5]. Although vitamin D analogs and calcimimetics may partially control the biochemical profile of these patients, parathyroidectomy (PTX), either subtotal (sPTX) or total (tPTX), with or without autotransplantation, remains the gold standard for definitive treatment [6, 7]. Meta-analyses demonstrate high cure rates (>95%) with both approaches, though recurrence is higher after sPTX, while permanent hypocalcemia is more frequent after tPTX without autograft [8, 9].

Autotransplantation sites include the sternocleidomastoid, forearm muscles (brachioradialis, brachialis), and tibialis anterior. Forearm implantation is generally favored for its accessibility, separation from the cervical operative field, ease of graft monitoring (tourniquet test, selective venous sampling), and safety of reoperation [10, 11]. The brachialis muscle, although less frequently used than the brachioradialis, provides a deeper, more protected site and preserves superficial vasculature for potential future dialysis access [12].

This report presents the first documented case in Greece of a renal transplant recipient with THPT who underwent tPTX with brachialis muscle autotransplantation, highlighting the surgical rationale, perioperative management, and postoperative outcome of this complex condition.

## Case presentation

A 72-year-old female presented with persistent biochemical and clinical features of hyperparathyroidism two years post-kidney transplantation. Despite treatment with cinacalcet (90 mg/day), her intact PTH (iPTH) levels remained between 300-400 pg/mL (reference range 10-65 pg/mL), and serum calcium ranged from 9.7-10.4 mg/dL (reference range 8.6-10.3 mg/dL). She reported fatigue and vague musculoskeletal discomfort that limited daily activities such as climbing stairs or carrying groceries but denied overt bone pain. There was no radiological or clinical evidence of nephrolithiasis.

The patient had a history of autosomal dominant polycystic kidney disease, which had progressed to ESRD requiring hemodialysis for six years prior to renal transplantation in April 2023. Her past medical history included arterial hypertension, chronic gastritis, hypothyroidism, and osteopenia.

Post-transplant immunosuppressive therapy consisted of tacrolimus (5 mg once daily) and mycophenolate mofetil (500 mg twice daily). For the first six months post-transplant, she also received valganciclovir and sulfamethoxazole-trimethoprim as prophylaxis against cytomegalovirus (CMV) and *Pneumocystis jirovecii *pneumonia (PCP), respectively. Concomitant medications included folic acid (5 mg daily), levothyroxine (62 μg daily), nebivolol (2.5 mg twice daily), amlodipine (5 mg daily), and magnesium aspartate hydrochloride trihydrate (1229.6 mg twice daily). She had also been treated with alendronate (70 mg weekly) for osteopenia.

Preoperative workup

Neck ultrasound and nuclear imaging identified multiple suspected hyperfunctioning parathyroid glands. Sestamibi scintigraphy revealed two foci of intense radiotracer uptake: one near the lower pole of the right thyroid lobe, further localized on single photon emission computed tomography and computed tomography (SPECT-CT) to a paraspinal position at the T1 vertebral level, and a second in contact with the posterior surface of the upper pole of the left thyroid lobe at the C6-C7 level. Additional areas of weaker uptake were observed inferior to the left lower pole and superior to the right upper pole of the thyroid gland.

**Figure 1:**
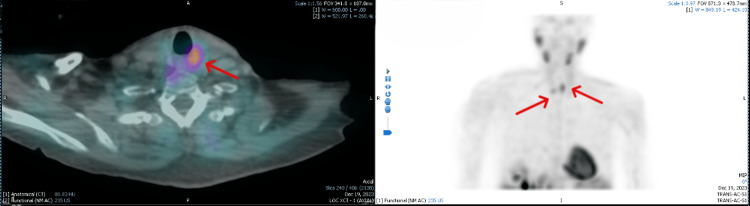
SPECT/CT demonstrating two foci of increased radiotracer uptake. Red arrows indicate the ectopic right superior parathyroid gland located in the paraesophageal region at the level of T1, and the left upper parathyroid gland posterior to the upper pole of the thyroid at C6-C7. Abbreviations: SPECT/CT: Single-Photon Emission Computed Tomography/Computed Tomography

**Figure 2:**
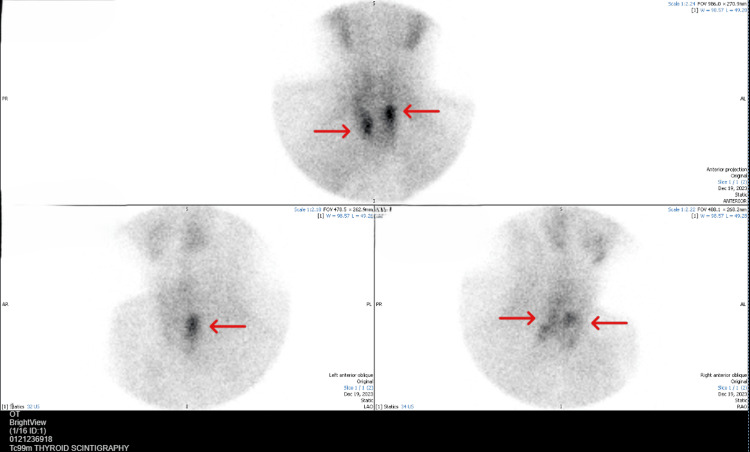
Tc-99m sestamibi thyroid scintigraphy. Arrows highlight focal tracer uptake corresponding to the ectopic right superior and left upper parathyroid glands, consistent with multiglandular THPT. Abbreviations: Tc-99m: Technetium-99m, THPT: tertiary hyperparathyroidism

Corresponding findings on contrast-enhanced 4-dimensional computed tomography (4D-CT) demonstrated a 1.3 × 0.5 cm elongated soft tissue lesion posterior to the left thyroid lobe and a 0.8 cm nodular lesion in the right paraoesophageal space, with a second smaller nodule, 0.3 cm, at a lower position. No other suspicious tissue was visualized around the thyroid. The imaging collectively supported the diagnosis of multiglandular parathyroid disease, including ectopic and posteriorly located lesions, consistent with THPT (Figures 1, 2).

Surgical intervention

**Figure 3:**
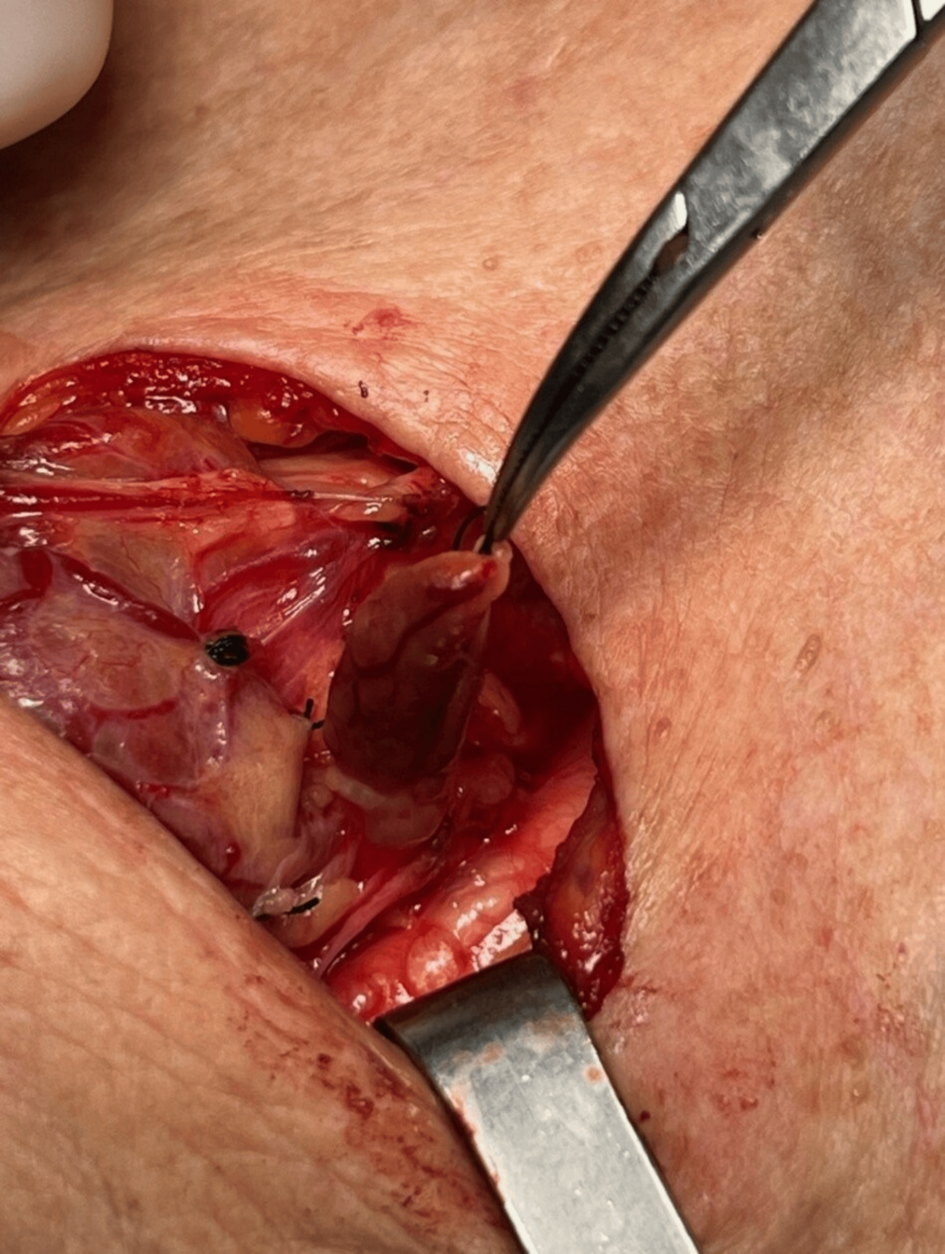
Intraoperative view of parathyroidectomy with gland dissection and identification of a large parathyroid gland.

On November 25, 2024, the patient underwent bilateral neck exploration with tPTX. All four parathyroid glands were identified and excised (Figure 3). The right superior gland was deeply embedded in the paraoesophageal fascia, necessitating careful dissection along the tracheoesophageal groove. Intraoperative frozen sections confirmed diffuse chief cell hyperplasia without evidence of adenomatous transformation or carcinoma. A summary of both the intraoperative frozen sections and final histological analysis is provided (Table 1).

**Table 1:** Histopathological findings from fast biopsy and full histological examination of parathyroid and adjacent tissues Abbreviations: g: grams, mm: millimeters, r: radius

Exam type	Anatomical location	Weight (g)	Size (mm)	Histology findings
Intraoperative frozen section	Right lower parathyroid	0.08	5×2×1.5	Parathyroid tissue, epithelial: fat ratio 60:40
Intraoperative frozen section	Right upper parathyroid	0.026	6×4×2	Parathyroid tissue, epithelial: fat ratio 85:15
Intraoperative frozen section	Left lower parathyroid	0.015	4×3×1	Parathyroid tissue, epithelial: fat ratio 70:30; includes thyroid tissue
Intraoperative frozen section	Left upper parathyroid	0.045	6×5×2	Parathyroid tissue, epithelial: fat ratio 80:20
Full exam	Tissues near thyroid	Not mentioned	r = 18 & r = 7	Fatty tissue only; lymphocyte infiltration; no parathyroid or lymph node elements
Full exam	Left upper parathyroid	0.8	18×14×4	Parathyroid hyperplasia; epithelial cells forming nests and microstructures; no malignancy
Full exam	Right upper parathyroid	0.612	23×9×3	Parathyroid hyperplasia; epithelial: fat ratio 90:10

**Figure 4:**
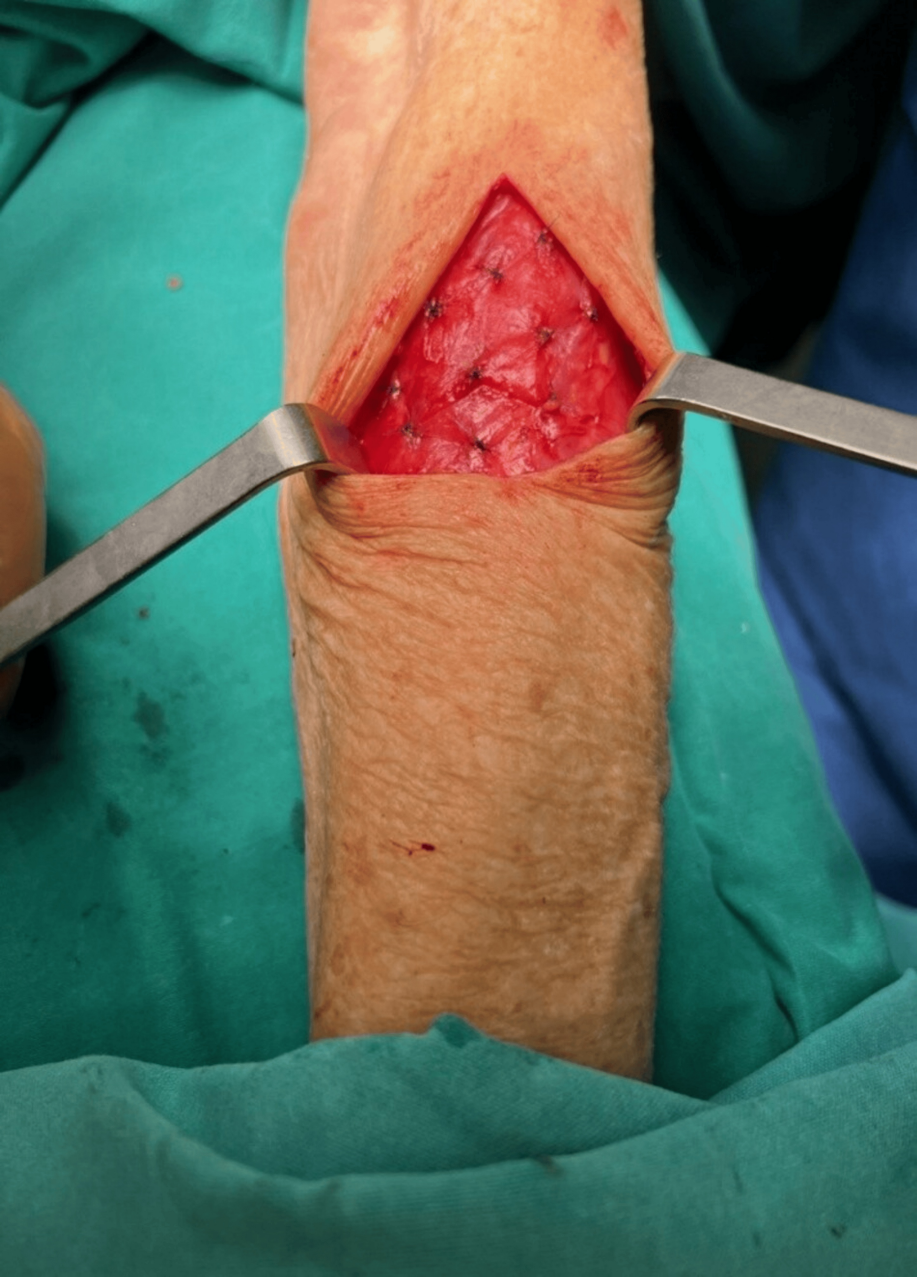
Parathyroid gland autotransplantation into the left brachialis muscle. Implantation site in the brachialis muscle post-placement of parathyroid tissue fragments, marked with titanium clips to enable future imaging and venous sampling.

From the two inferior glands (showing less pronounced hyperplasia), parathyroid tissue was selected for autotransplantation. Using standardized techniques, 15 parathyroid tissue fragments (~1 mm³ each) were implanted into a prepared pocket within the left brachialis muscle at a depth of 2-3 mm (Figure 4). The site was demarcated with titanium clips to allow for future imaging and targeted venous sampling if necessary.

Intraoperative and immediate postoperative biochemical monitoring

The baseline iPTH was 350 pg/mL. Ten minutes following the excision of all glands, it was 40 pg/mL. Twelve hours postoperatively, it was <5 pg/mL.

The patient received six ampoules of intravenous (IV) calcium gluconate followed by oral calcium carbonate supplementation (1 g twice per day) and alfacalcidol (1 mcg per day) for seven days due to transient postoperative hypocalcemia. No signs of tetany or clinical hypocalcemia were noted. Cinacalcet was permanently discontinued.

Postoperative course and complications

On postoperative day 3, the patient developed mild painless swelling and violaceous discoloration of the left forearm and fingers. Venous sampling from the ipsilateral arm revealed a markedly elevated iPTH of 980 pg/mL, while systemic levels remained <5 pg/mL. This confirmed the diagnosis of localized venous hyperparathyroidism due to early graft hormone secretion, a benign, self-limiting phenomenon that resolved spontaneously within 15 days. Follow-up postoperative laboratory results are summarized in Table 2.

**Table 2:** Follow-up postoperative laboratory results. Reference range: iPTH:10-65 pg/mL, Calcium: 9.7-10.4 mg/dL Abbreviations: iPTH: intact parathyroid hormone, pg/mL: picograms per milliliter, mg/dL: milligrams per deciliter

Postoperative Time	iPTH (pg/mL)	Calcium (mg/dL)
Day 1	<5	8.4
3 months	109	8.9
5 months	140	9.1
10 months	57	9.3

Renal graft function remained stable throughout follow-up (creatinine: 1.0-1.1 mg/dL with reference range of 0.5-1.1 mg/dL). Six months after the parathyroidectomy, a follow-up bone densitometry confirmed osteoporosis. Alendronate was discontinued and replaced with denosumab (60 mg subcutaneously every six months) in combination with cholecalciferol (25,000 IU weekly). Serum calcium levels remained mildly below the upper normal range but stable, and calcium supplementation was withheld. The patient remained clinically asymptomatic throughout follow-up.

## Discussion

Persistent hyperparathyroidism following successful renal transplantation is a well-recognized consequence of longstanding SHPT, particularly in patients with delayed access to renal replacement therapy or those with suboptimal medical management prior to transplantation [13, 14]. Chronic stimulation of the parathyroid glands leads to hyperplastic transformation, often progressing to nodular hyperplasia, a histological hallmark of THPT. In such cases, the parathyroid glands acquire functional autonomy, rendering them resistant to normal feedback inhibition by serum calcium or calcimimetic agents [1, 3, 5].

Reported pre-transplant predictors of THPT include elevated PTH levels >300 pg/mL, prolonged dialysis duration, and prior calcimimetic therapy [2, 13]. In these patients, spontaneous regression following transplantation is unlikely, and early surgical referral within 6-12 months after grafting is increasingly advocated [14].

Comprehensive preoperative imaging is essential not only to confirm multiglandular involvement but also to detect ectopic parathyroid tissue, which can significantly influence surgical planning [15]. While conventional ultrasound may fail to identify deep or ectopically located glands, Technetium-99m sestamibi scintigraphy provides functional information about hyperactive tissue [16]. More recently, 4D-CT has emerged as a superior modality, particularly for detecting ectopic glands located in paraoesophageal, mediastinal, or retroesophageal spaces [15, 16]. In our patient, the discovery of an ectopic right superior gland in the paraoesophageal groove prompted the decision to proceed with a tPTX rather than a subtotal resection, ensuring definitive excision of all hyperfunctional tissue.

Both sPTX and tPTX with autotransplantation are considered effective strategies. A 2024 meta-analysis of 28 studies found no statistically significant differences in cure, recurrence, or complication rates between the two approaches [8]. However, the decision must be guided by individual patient factors and clinical context. sPTX preserves cervical parathyroid tissue, but recurrence from the remnant is well documented [9] and often necessitates reoperation, which carries substantial risk due to scar tissue and proximity to the recurrent laryngeal nerves [17]. Conversely, tPTX without autotransplantation nearly eliminates recurrence but increases the risk of permanent hypoparathyroidism, requiring lifelong supplementation [7]. tPTX with autotransplantation balances these outcomes, allowing residual parathyroid function while enabling easier monitoring and re-excision if necessary [11].

Rationale for brachialis muscle autotransplantation

Forearm implantation is the preferred extracephalic site because it provides easy access, clear separation from the cervical field, and facilitates postoperative monitoring. The brachialis muscle, although less commonly utilized than the brachioradialis, offers greater depth, mechanical protection, and preservation of superficial forearm vasculature, advantages particularly relevant for renal transplant recipients who may require future dialysis access. Long-term graft survival rates of 85-92% have been reported for forearm autotransplantation [10], whereas tibialis anterior grafting offers comparable biochemical outcomes (86-96%) [11].

Our case illustrates these benefits. Postoperative iPTH normalized (57 pg/mL at 10 months) with stable calcium and renal function, confirming durable graft viability. Importantly, localized venous hyperparathyroidism, manifesting as mild forearm swelling and discoloration, resolved spontaneously within two weeks, demonstrating benign early graft activity.

Our choice was further supported by two practical considerations. First, postoperative PTH elevation can be easily investigated using the "tourniquet test." In this test, venous return from the grafted forearm is occluded for 6-7 minutes, and serum PTH is then remeasured. A significant drop in systemic PTH indicates that the autograft is the source of hormone secretion, rather than residual parathyroid tissue in the neck. This functional test offers a simple and practical method to localize persistent parathyroid activity and guide further management [11]. Second, in the event of recurrence, reoperation at the forearm graft site is significantly easier and safer than reoperating in the cervical region [17].

Anatomical, surgical safety, and cosmetic considerations

Autotransplantation into the brachialis muscle offers important anatomical separation from the cervical operative field, preserving the integrity of vital neck structures during potential future reoperations. This is particularly beneficial in patients with prior neck surgeries, where scar tissue, adhesions, and altered anatomy elevate the risk of complications such as recurrent laryngeal or hypoglossal nerve injury [17]. The deeper plane of the brachialis muscle also provides mechanical protection for the graft, reducing susceptibility to external trauma compared to the more superficial brachioradialis.

Forearm implantation also facilitates selective venous sampling from the ipsilateral arm, enabling precise evaluation of graft function independent of systemic PTH levels. This diagnostic utility is especially valuable in the early postoperative period or when recurrence is suspected [3]. In our patient, this approach confirmed localized hormone production, as iPTH levels from the grafted arm reached 980 pg/mL compared to undetectable systemic levels.

From a patient-centered perspective, the forearm is generally more acceptable cosmetically than a revisited neck incision and provides easier access for both imaging and biopsy. The brachialis muscle’s depth also reduces exposure to minor trauma compared with the more superficial brachioradialis [10]. These advantages may improve patient comfort and acceptance of postoperative follow-up.

Challenges and complications

Despite these advantages, forearm autotransplantation is not without drawbacks. Localized venous hyperparathyroidism, as demonstrated in our case, is an underrecognized but important phenomenon. Early after grafting, the local venous effluent may contain disproportionately high PTH concentrations, potentially leading to misinterpretation of systemic recurrence or transient symptoms such as limb edema and discoloration [11]. Awareness of this localized hormonal flux is crucial to prevent unnecessary interventions.

Furthermore, the long-term success of parathyroid autotransplantation depends on several variables:

Graft Selection: Diffusely hyperplastic tissue is generally preferred over nodular tissue, which is associated with higher recurrence rates due to monoclonal cellular proliferation [9].

Vascularization and Implantation Technique: Ensuring adequate contact with well-perfused muscle fibers and avoiding overpacking of graft fragments is essential to support neovascularization and viability [9, 10]. In our case, 15 uniform 1 mm³ fragments were spaced within a vascular plane at 2-3 mm depth, an established technique aimed at promoting reliable engraftment.

Host Immunologic Environment: In transplant recipients, immune modulation may impact graft take and function, although autologous implantation is generally well tolerated and unaffected by systemic immunosuppression [11].

Clinical implications

Despite strong evidence for surgical efficacy, under-treatment of THPT remains common. Large cohort studies demonstrate that fewer than 10% of post-transplant patients with THPT undergo PTX, despite well-documented associations with graft dysfunction, bone disease, and cardiovascular events when left untreated [13, 14]. This highlights the need for greater awareness among nephrologists and transplant teams regarding timely surgical referral.

Our case illustrates that tPTX with brachialis muscle autotransplantation can achieve durable biochemical control while offering distinct advantages in monitoring, re-exploration safety, and preservation of forearm vasculature for potential future dialysis access, providing practical insight into an uncommon but effective surgical technique.

## Conclusions

Total parathyroidectomy with autotransplantation into the brachialis muscle is a safe, effective, and technically refined option for the management of THPT, particularly in patients with ectopic glands, prior cervical surgery, or anatomically complex presentations. This approach allows for reliable graft monitoring, avoids the morbidity associated with re-operative neck exploration, and preserves functional parathyroid tissue in a controlled, accessible location. In our patient, postoperative biochemical remission was achieved with normalization of iPTH (57 pg/mL at 10 months), stable calcium and renal function, confirming durable graft function. Histopathology demonstrated diffuse chief cell hyperplasia without malignancy. Transient localized venous hyperparathyroidism occurred in the grafted forearm during the early postoperative period and resolved spontaneously within 15 days. Careful graft selection, favoring non-nodular tissue, is essential to minimize recurrence and ensure long-term graft stability. This case not only illustrates the importance of individualized surgical strategy and interdisciplinary care but also represents the first documented instance of brachialis muscle autotransplantation for THPT in Greece, contributing a novel insight to the evolving surgical management of this condition.
